# Mechanisms of *Candida albicans* Trafficking to the Brain

**DOI:** 10.1371/journal.ppat.1002305

**Published:** 2011-10-06

**Authors:** Yaoping Liu, Rahul Mittal, Norma V. Solis, Nemani V. Prasadarao, Scott G. Filler

**Affiliations:** 1 Division of Infectious Diseases, Los Angeles Biomedical Research Institute at Harbor-UCLA Medical Center, Torrance, California, United States of America; 2 Division of Infectious Diseases, The Saban Research Institute, Childrens Hospital Los Angeles, California, United States of America; 3 Keck School of Medicine, University of Southern California, Los Angeles, California, United States of America; 4 David Geffen School of Medicine at UCLA, Los Angeles, California, United States of America; University of Toronto, Canada

## Abstract

During hematogenously disseminated disease, *Candida albicans* infects most organs, including the brain. We discovered that a *C. albicans vps51*Δ/Δ mutant had significantly increased tropism for the brain in the mouse model of disseminated disease. To investigate the mechanisms of this enhanced trafficking to the brain, we studied the interactions of wild-type *C. albicans* and the *vps51*Δ/Δ mutant with brain microvascular endothelial cells *in vitro*. These studies revealed that *C. albicans* invasion of brain endothelial cells is mediated by the fungal invasins, Als3 and Ssa1. Als3 binds to the gp96 heat shock protein, which is expressed on the surface of brain endothelial cells, but not human umbilical vein endothelial cells, whereas Ssa1 binds to a brain endothelial cell receptor other than gp96. The *vps51*Δ/Δ mutant has increased surface expression of Als3, which is a major cause of the increased capacity of this mutant to both invade brain endothelial cells *in vitro* and traffic to the brain in mice. Therefore, during disseminated disease, *C. albicans* traffics to and infects the brain by binding to gp96, a unique receptor that is expressed specifically on the surface of brain endothelial cells.

## Author Summary

During hematogenously disseminated infection, the fungus *Candida albicans* is carried by the bloodstream to virtually all organs in the body, including the brain. *C. albicans* infection of the brain is a significant problem in premature infants with disseminated candidiasis. To infect the brain, *C. albicans* must adhere to and invade the endothelial cells that line cerebral blood vessels. These endothelial cells express unique proteins on their surface that are not expressed by endothelial cells of other vascular beds. Here, we show that *C. albicans* infects the brain by binding to gp96, a heat shock protein that is uniquely expressed on the surface of brain endothelial cells. Gp96 is bound by the *C. albicans* Als3 invasin, which induces the uptake of this organism by brain endothelial cells. The *C. albicans* Ssa1 invasin also mediates fungal uptake by brain endothelial cells, but does so by binding to a receptor other than gp96. Thus, during hematogenously disseminated infection, *C. albicans* traffics to and infects the brain by binding to gp96, a receptor that is expressed specifically on the surface of brain endothelial cells.

## Introduction

Hematogenously disseminated candidiasis is a serious disease that remains associated with approximately 35% mortality, even with currently available treatment, and *Candida albicans* is the infecting organism in approximately 50% of patients [1], [2]. During this disease, *C. albicans* is carried by the bloodstream to virtually all organs of the body, including the brain. Although candidal infection of the brain may not be clinically evident in adults with disseminated candidiasis, it is frequently found at autopsy in patients who die of this disease [3]. Even more importantly, candidal brain infection, especially meningitis, is a significant problem in premature infants who have risk factors for disseminated candidiasis, even in the absence of detectable candidemia [4], [5].

To invade the brain parenchyma, blood-borne *C. albicans* cells must adhere to and traverse the endothelial cell lining of the blood vessels within the central nervous system. Brain endothelial cells are significantly different from those lining systemic blood vessels. For example, they have tight junctions that are not present in the endothelial cells in other vascular beds. Forming the blood brain barrier, brain endothelial cells restrict the diffusion of large or hydrophilic molecules into the central nervous system, while allowing the diffusion of small hydrophobic molecules [6]. More importantly, some microbial pathogens, such as *Neisseria meningitidis*, *Streptococcus pneumonia*, *Escherichia coli* K1, and *Cryptococcus neoformans* have an enhanced capacity to adhere to and invade human brain microvascular endothelial cells (HBMECs), which enables them to preferentially infect the central nervous system via the hematogenous route [7]–[11]. Thus, these pathogens can exploit the unique characteristics of HBMECs to specifically infect the brain.

Studies using human umbilical vein endothelial cells (HUVECs) as representative systemic endothelial cells have demonstrated that *C. albicans* adheres to, invades, and damages these cells *in vitro* [12], [13]. One mechanism by which *C. albicans* invades these cells is by stimulating its own endocytosis, which is induced when the *C. albicans* invasins, Als3 and Ssa1, bind to receptors such as N-cadherin and HER2 on the endothelial cell surface [14]–[17]. *C. albicans* yeast and hyphae can also invade HBMECs by inducing their own endocytosis [18], [19]. However, the mechanism by which this pathogen invades these endothelial cells and infects the brain is poorly understood.

Recently we discovered that *C. albicans VPS51* is up-regulated by contact with HUVECs *in vitro*, and that a *vps51/vps51* insertion mutant is defective in damaging these endothelial cells [20]. In *Saccharomyces cerevisiae*, Vps51 is known to bind to the Vps52/53/54 complex and is required for the retrograde transport of proteins from endosomes to the late Golgi [21], [22]. Although the function of Vps51 in *C. albicans* has not been studied in detail, the *vps51/vps51* insertion mutant has a fragmented vacuole, similar to the corresponding *S. cerevisiae* mutant [20]–[22]. Thus, Vps51 likely plays a role in protein trafficking in *C. albicans*.

In the current study, we investigated how deletion of *VPS51* affects the virulence of *C. albicans* during hematogenous infection. We found that the *vps51*Δ/Δ null mutant exhibits a preferential tropism for the brain. This tropism is mediated in part by the enhanced exposure of Als3 on the surface of the *vps51*Δ/Δ mutant, which binds to gp96 on the surface of HBMECs and mediates invasion of these endothelial cells. We further discovered that gp96 functions as a receptor for wild-type *C. albicans* on HBMECs, but not HUVECs, indicating that this organism invades the central nervous system by binding to a receptor that is expressed specifically on HBMECs.

## Results

### Deletion of *VPS51* causes reduced mortality and decreased kidney and liver fungal burden during hematogenously disseminated candidiasis

**Figure 1:**
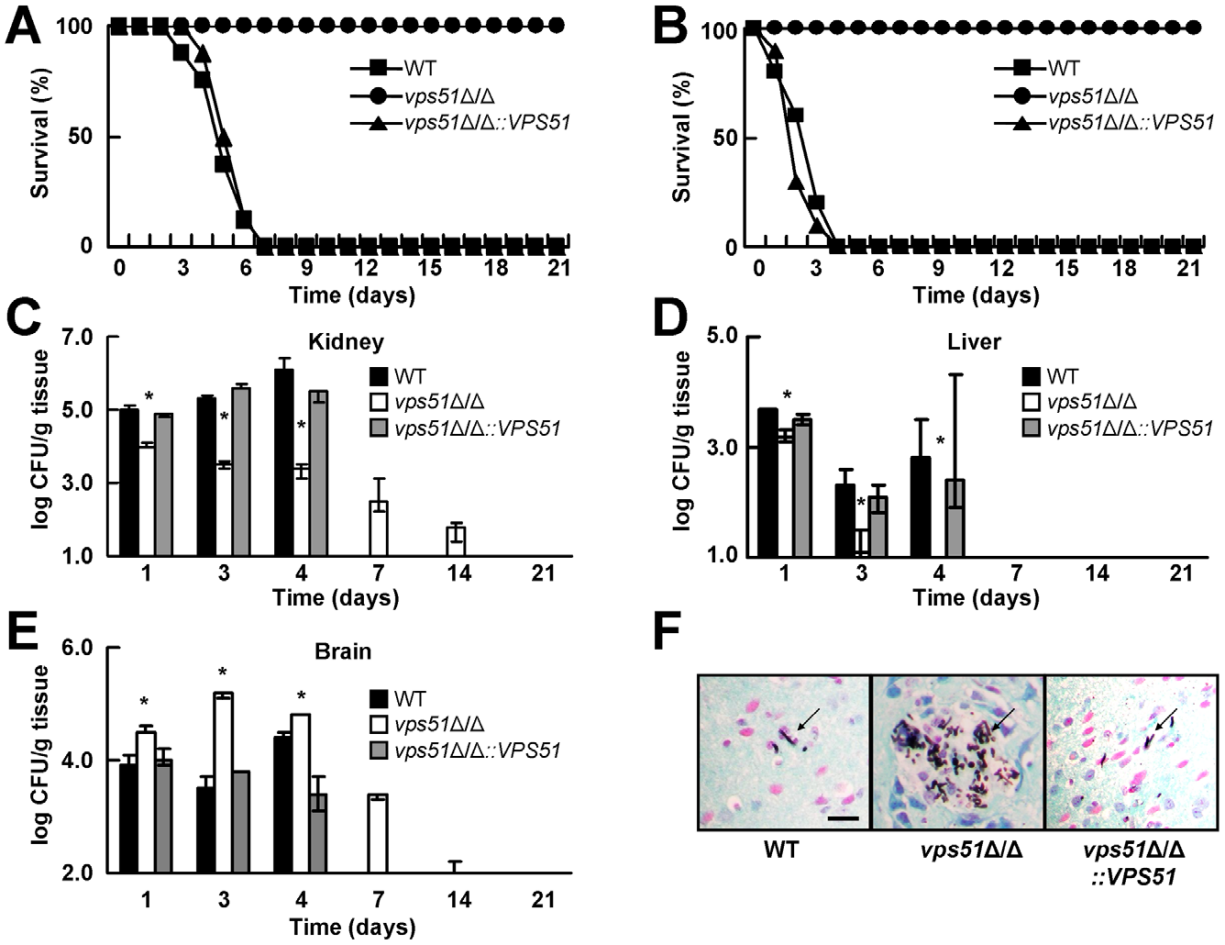
A *vps51*Δ/Δ mutant has attenuated overall virulence, but increased brain tropism during hematogenously disseminated candidiasis. (A and B) Survival of mice after intravenous inoculation with 5×10^5^ (A) or 3×10^6^ (B) yeast phase cells of the indicated strains of *C. albicans* (n = 10 mice per strain). (C – E) Fungal burden of the kidneys (C), liver (D), and brain (E) of mice at the indicated times after inoculation with 5×10^5^ cells of the various strains. Results from days 1, 4, 7, 14, and 21 are median ± interquartile range of a single experiment with 7 mice per strain at each time point. Data from day 3 are the combined results from two experiments, each with 6–7 mice per strain. Only mice infected with the *vps51*Δ/Δ mutant were analyzed at the 7, 14, and 21 day time points because all of the mice infected with the other *C. albicans* strains had died. At these later time points, the absence of a bar indicates that the fungal burden was below the limits of detection. *p<0.01 compared to the wild-type (WT) and *vps51*Δ/Δ*+*p*VPS51* complemented strains by the Wilcoxon Rank Sum Test. (F). Histopathology of the hypothalamus of mice after 3 days of infection with the indicated strains. Sections were stained with Gomori methenamine silver. Scale bar 10 µm. Arrows indicate the organisms.

To investigate the role of Vps51 in the virulence of *C. albicans*, we inoculated mice via the lateral tail vein with a wild-type strain, a *vps51*Δ/Δ mutant, and a *vps51*Δ/Δ+p*VPS51* complemented strain and then monitored their survival over time. We found that all mice infected with the *vps51*Δ/Δ mutant survived for the entire 21-day observation period, whereas all mice infected with the wild-type strain died within 7 days after inoculation (Figure 1A). Complementing the *vps51*Δ/Δ mutant with an intact copy of *VPS51* restored its virulence to wild-type levels, thus confirming that Vps51 is required for the maximal virulence of *C. albicans*. The greatly reduced virulence of the *vps51*Δ/Δ mutant was further verified by infecting mice with a 6-fold higher inoculum. As expected, mice infected with the wild-type strain at this higher inoculum died rapidly, with a median survival of only 3 days (Figure 1B). However, all mice infected with the *vps51*Δ/Δ mutant still survived. Therefore, Vps51 is necessary for the full virulence of *C. albicans*.

The mouse model of hematogenous disseminated candidiasis mimics many aspects of this disease in humans, particularly the formation of microabscesses in most organs [23], [24]. We therefore investigated the effects of deleting *VPS51* on organ fungal burden. During the first 4 days of infection, the kidneys and livers of mice infected with the *vps51*Δ/Δ mutant contained significantly fewer organisms than those of mice infected with either the wild-type or *vps51*Δ/Δ*+*p*VPS51* complemented strain (Figure 1C and D). Furthermore, the kidney fungal burden of mice infected with the *vps51*Δ/Δ mutant progressively declined after the first day of infection. In contrast, the kidney fungal burden of mice infected with the wild-type and *vps51*Δ/Δ*+*p*VPS51* complemented strains progressively increased for the first 4 days post-infection, after which these mice began to die. These results further demonstrate that the overall virulence of the *vps51*Δ/Δ mutant is decreased.

### Absence of Vps51 or Vps53 results in increased brain fungal burden

A surprising result was that during the first 4 days of infection, the brain fungal burden of mice infected with the *vps51*Δ/Δ mutant was significantly greater than that of mice infected with either the wild-type or *vps51*Δ/Δ*+*p*VPS51* complemented strain (Figure 1E). Indeed, after 3 days of infection, the brains of mice infected with the *vps51*Δ/Δ mutant contained a median of 50-fold more organisms than those of mice infected with the wild-type strain. Despite having a high brain fungal burden, the mice infected with the *vps51*Δ/Δ mutant did not appear to be sick and had no obvious signs of neurological disease. Moreover, beginning on the fourth day of infection, these mice progressively cleared the organisms from their central nervous system. These results suggest that while the overall virulence of the *vps51*Δ/Δ mutant is decreased, it has a distinct tropism for the brain.

We verified these quantitative culture results by performing histopathologic analysis of the brains of the infected mice. Foci containing multiple organisms were visible in the brains of mice infected with the *vps51*Δ/Δ mutant, especially in the hippocampus (Figure 1F). In sharp contrast, only rare organisms were visible in the brains of the mice infected with either the wild-type or *vps51*Δ/Δ*+*p*VPS51* complemented strains, and these organisms were typically either solitary or in pairs.

**Figure 2:**
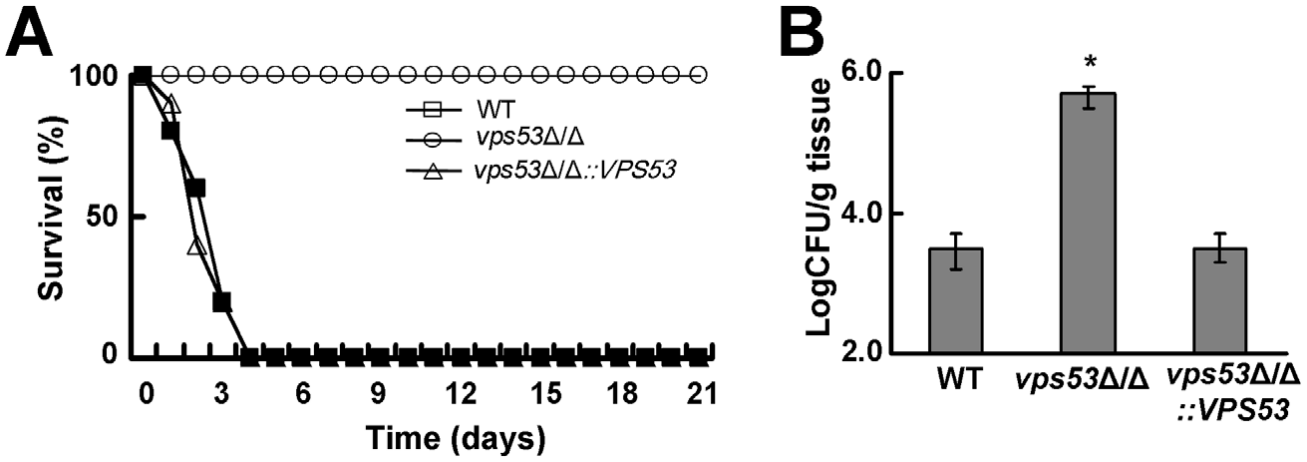
Deletion of *VPS53* causes reduced mortality and increased brain fungal burden during hematogenously disseminated candidiasis. (A) Survival of mice after intravenous inoculation of with 3×10^6^ yeast phase cells of the indicated strains. 10 mice were infected with each strain. (B) Increased brain fungal burden of mice 3 days after intravenous inoculation of 5×10^5^ cells per strain. Results are the median ± interquartile ranges of 7 mice per strain. *p<0.01 compared to the wild-type and *vps53*Δ/Δ+p*VPS53* complemented strains.

To determine if another member of the Vps51/52/53/54 complex is required for maximal virulence and enhanced brain tropism, we constructed and analyzed a *vps53*Δ/Δ mutant. This strain also caused no mortality in mice following tail vein inoculation. (Figure 2A) Furthermore, it accumulated at significantly higher levels in the brain than did the wild-type and *vps53*Δ/Δ+p*VPS53* complemented strains (Figure 2B). Collectively, these results demonstrate that the Vps51/52/53/54 complex plays a key role in virulence, and that *C. albicans* strains that lack components of this complex preferentially infect the brain.

### The *vps51*Δ/Δ mutant has an increased capacity to adhere to and invade HBMECs

**Figure 3:**
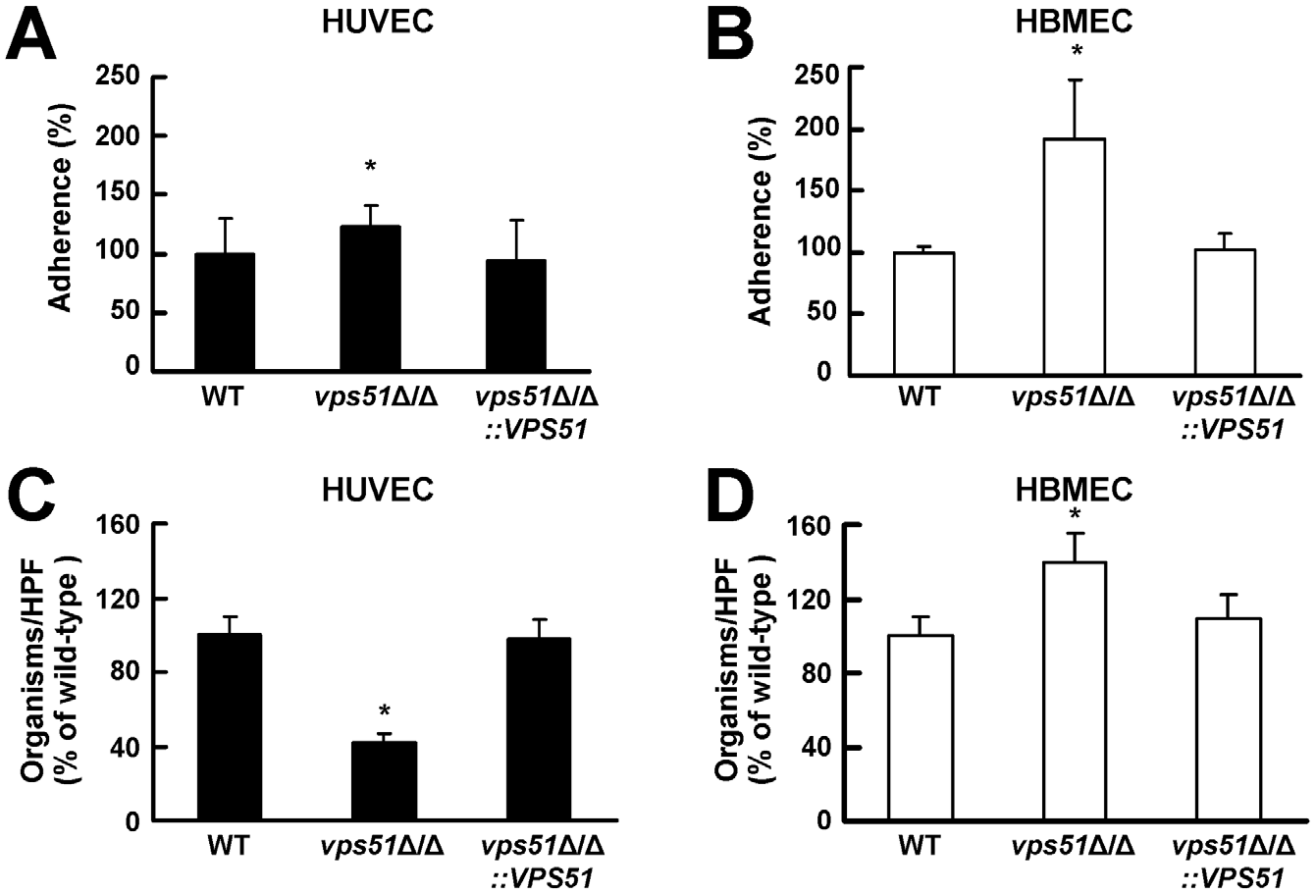
The *vps51*Δ/Δ mutant interacts differently with human umbilical vein endothelial cells (HUVECs) versus human brain microvascular endothelial cells (HBMECs). (A and B) Adherence of germ tubes of the indicated *C. albicans* strains to HUVECs (A) and HBMECs (B). (C and D) Endocytosis of hyphae of the indicated strains by HUVECs (C) and HBMECs (D). The results are expressed as a percentage of the wild-type strain and are the mean ± SD of 3 experiments, each performed in triplicate. The mean adherence of the wild-type strain to HUVECs and HBMECs was 47% and 28%, respectively. The mean number of wild-type cells endocytosed by HUVECs and HBMECs was 109 and 35 organisms per 10 high-powered fields (HPF), respectively. *p<0.01 compared to both the wild-type strain and the *vps51*Δ/Δ*+*p*VPS51* complemented strain by Analysis of Variance.

To cross the endothelial cell lining of the vasculature, *C. albicans* must first adhere to these endothelial cells and then invade through them. We hypothesized that the *vps51*Δ/Δ mutant had increased capacity to infect the brain because it preferentially adhered to and invaded the unique endothelial cells that line the blood vessels of central nervous system. To test this hypothesis, we compared the interactions of this mutant with HUVECs and HBMECs *in vitro*. We found that the adherence of the *vps51*Δ/Δ mutant to HUVECs was increased by only 22% compared to the wild-type strain (Figure 3A). However, the adherence of this mutant to HBMECs was increased by 95% (Figure 3B). There was an even greater difference in the capacity the *vps51*Δ/Δ mutant to induce its own endocytosis by HUVECs compared to HBMECs. The endocytosis of the *vps51*Δ/Δ mutant by HUVECs was 58% lower than that of the wild-type strain (Figure 3C). In contrast, the endocytosis of this mutant by HBMECs was 39% higher than the wild-type strain (Figure 3D). Complementing the *vps51*Δ/Δ mutant with an intact copy of *VPS51* restored its interactions with both types of endothelial cells to wild-type levels. The increased capacity of the *vps51*Δ/Δ mutant to adhere to and invade HBMECs compared to HUVECs provides a likely explanation for the enhanced tropism of this mutant for the brain.

### Gp96 mediates *C. albicans* invasion of HBMECs

**Figure 4:**
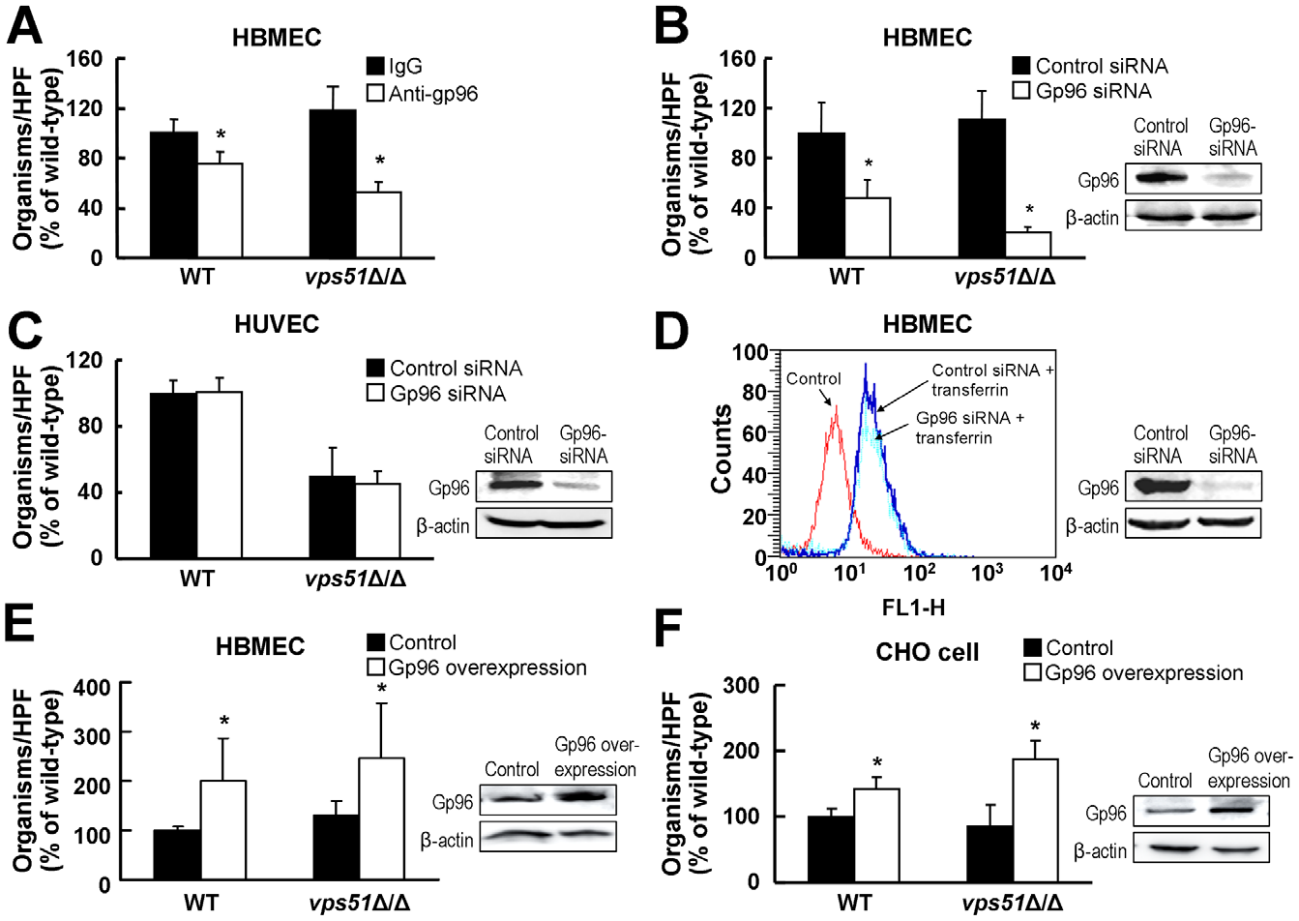
*C. albicans* endocytosis by HBMECs is mediated by gp96. (A) Reduced HBMEC endocytosis of wild-type and *vps51*Δ/Δ mutant strains of *C. albicans* by an anti-gp96 antibody. (B and C) Knockdown of gp96 by siRNA inhibits the endocytosis of *C. albicans* by HBMECs (B), but not by HUVECs (C). (D) Gp96 knockdown has no effect on transferrin endocytosis by HBMECs. (E and F) Overexpression of human gp96 in HBMECs (E) and Chinese Hamster Ovary (CHO) cells (F) results in increased endocytosis of *C. albicans*. The results are expressed as a percentage of the wild-type strain and are the mean ± SD of 3 experiments, each performed in triplicate. The mean number of wild-type cells endocytosed by control HBMECs, HUVECs, and CHO cells was 41, 49, and 48 organisms per 10 HPF, respectively. *p<0.01 compared to control strains. Images to the right of the graphs are of representative immunoblots showing the effects of the interventions on total gp96 and β-actin protein levels in the cells.

Next, we sought to identify the HBMEC receptor for both wild-type *C. albicans* and the *vps51*Δ/Δ mutant. HBMECs are known to express high amounts of the heat shock protein, gp96 on their cell surface, whereas HUVECs do not [9]. Furthermore, gp96 functions as an HBMEC-specific receptor for *E. coli* K1 strains that cause neonatal meningitis [9]. We used multiple complementary approaches to evaluate whether gp96 expression is required for *C. albicans* to invade HBMECs. First, we tested the capacity of an anti-gp96 antibody to inhibit HBMEC endocytosis of *C. albicans*. This antibody reduced the endocytosis of wild-type *C. albicans* by 24% and the *vps51*Δ/Δ mutant by 48% (Figure 4A). Second, we determined the effects of siRNA-mediated knockdown of gp96 on endocytosis. HBMECs transfected with gp96 siRNA endocytosed 52% fewer wild-type *C. albicans* cells and 82% fewer *vps51*Δ/Δ cells than did HBMECs transfected with control siRNA (Figure 4B). Importantly, the effect of gp96 knockdown on endocytosis was specific for HBMECs because knockdown of gp96 in HUVECs had no effect on their capacity to endocytose *C. albicans* (Figure 4C). Also, knockdown of gp96 did not inhibit HBMECs endocytosis of transferrin (Figure 4D), demonstrating that reducing gp96 protein levels did not cause a global decrease in receptor-mediated endocytosis. Collectively, these results indicate that gp96 is required for maximal HBMEC endocytosis of both wild-type *C. albicans* and the *vps51*Δ/Δ mutant.

To further explore these findings, we investigated the effects of overexpressing gp96 on the endocytosis of *C. albicans*. We found that overexpression of gp96 in HBMECs enhanced the endocytosis of the wild-type strain and the *vps51*Δ/Δ mutant by 100% and 115%, respectively (Figure 4E). Similarly, heterologous expression of human gp96 in Chinese hamster ovary (CHO) cells resulted in a 42% increase in the endocytosis of wild-type *C. albicans* and a 102% increase in the endocytosis of the *vps51*Δ/Δ mutant compared to control CHO cells transfected with the empty vector (Figure 4F). Therefore, these combined results demonstrate that gp96 functions as an HBMEC receptor that mediates the endocytosis of both wild-type *C. albicans* and the *vps51*Δ/Δ mutant.

### *C. albicans* Als3 and Ssa1 mediate HBMEC endocytosis *in vitro*

**Figure 5:**
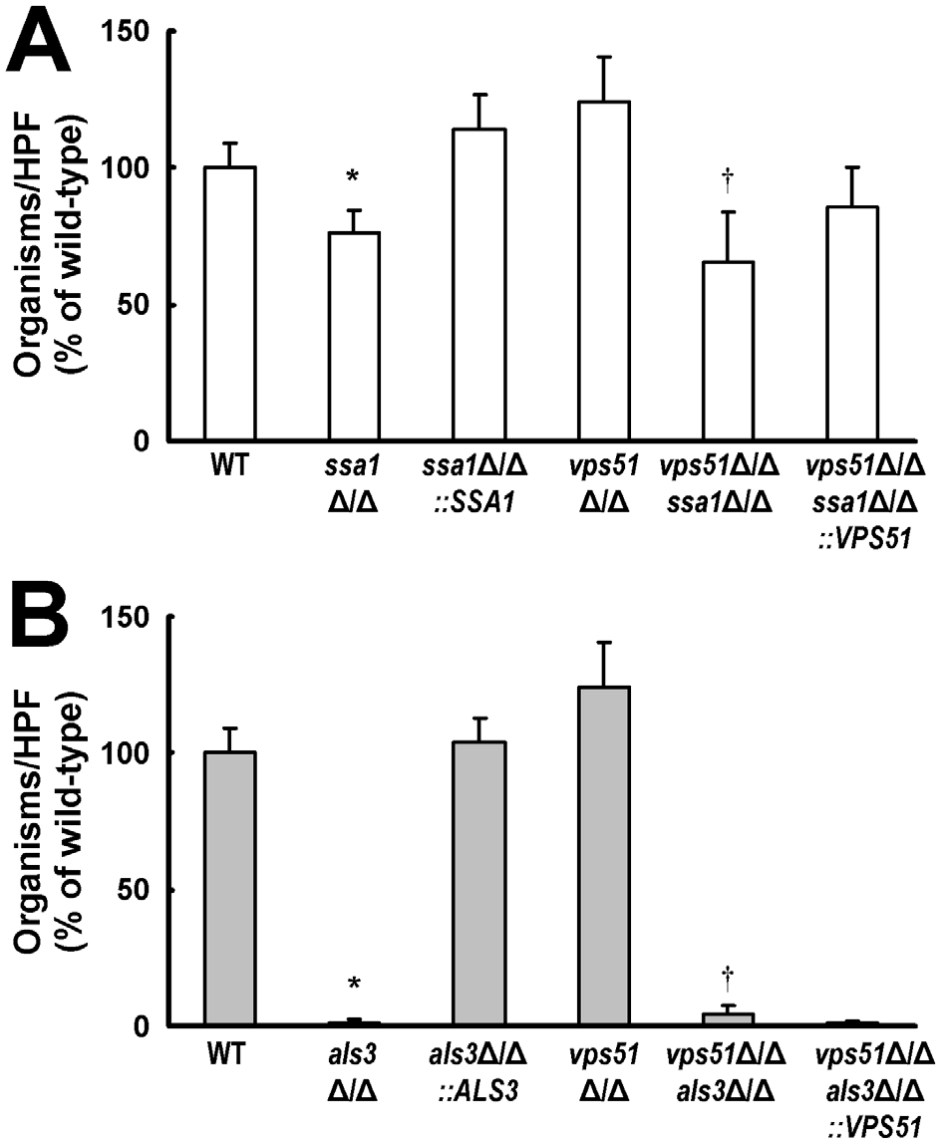
Deletion of *SSA1* or *ALS3* reduces HBMEC endocytosis of *C. albicans.* (A and B) Endocytosis of the indicated strains of *C. albicans* by HBMECs. The results are expressed as a percentage of the wild-type strain and are the mean ± SD of 3 experiments, each performed in triplicate. A mean 65 of wild-type cells per HPF was endocytosed by the HBMECs. *p<0.01 compared to the wild-type strain; †*p*<0.01 compared to the *vps51*Δ/Δ mutant.

Our previous studies revealed that the *C. albicans* proteins Ssa1 and Als3 function as invasins that induce the endocytosis of this organism by HUVECs [14], [15]. To investigate the roles of these fungal proteins in HBMEC invasion, we analyzed *ssa1*Δ/Δ and *als3*Δ/Δ single mutants, as well as *vps51*Δ/Δ *ssa1*Δ/Δ and *vps51*Δ/Δ *als3*Δ/Δ double mutants. Approximately 30% fewer hyphae of the *ssa1*Δ/Δ single mutant were endocytosed by HBMECs as compared to wild-type parent strain and the *ssa1*Δ/Δ*+*p*SSA1* complemented strain (Figure 5A). Similarly, the endocytosis of the *vps51*Δ/Δ *ssa1*Δ/Δ double mutant was significantly lower than the *vps51*Δ/Δ single mutant. Thus, Ssa1 is required for the maximal endocytosis of both wild-type and *vps51*Δ/Δ mutant strains of *C. albicans* by HBMECs *in vitro*.

Als3 played a greater role than Ssa1 in stimulating the endocytosis of *C. albicans* by HBMECs *in vitro*. Both the *als3*Δ/Δ single mutant and the *vps51*Δ/Δ *als3*Δ/Δ double mutant were endocytosed extremely poorly by these endothelial cells (Figure 5B), indicating that Als3 is essential for the endocytosis of *C. albicans* by HBMECs *in vitro*.

**Figure 6:**
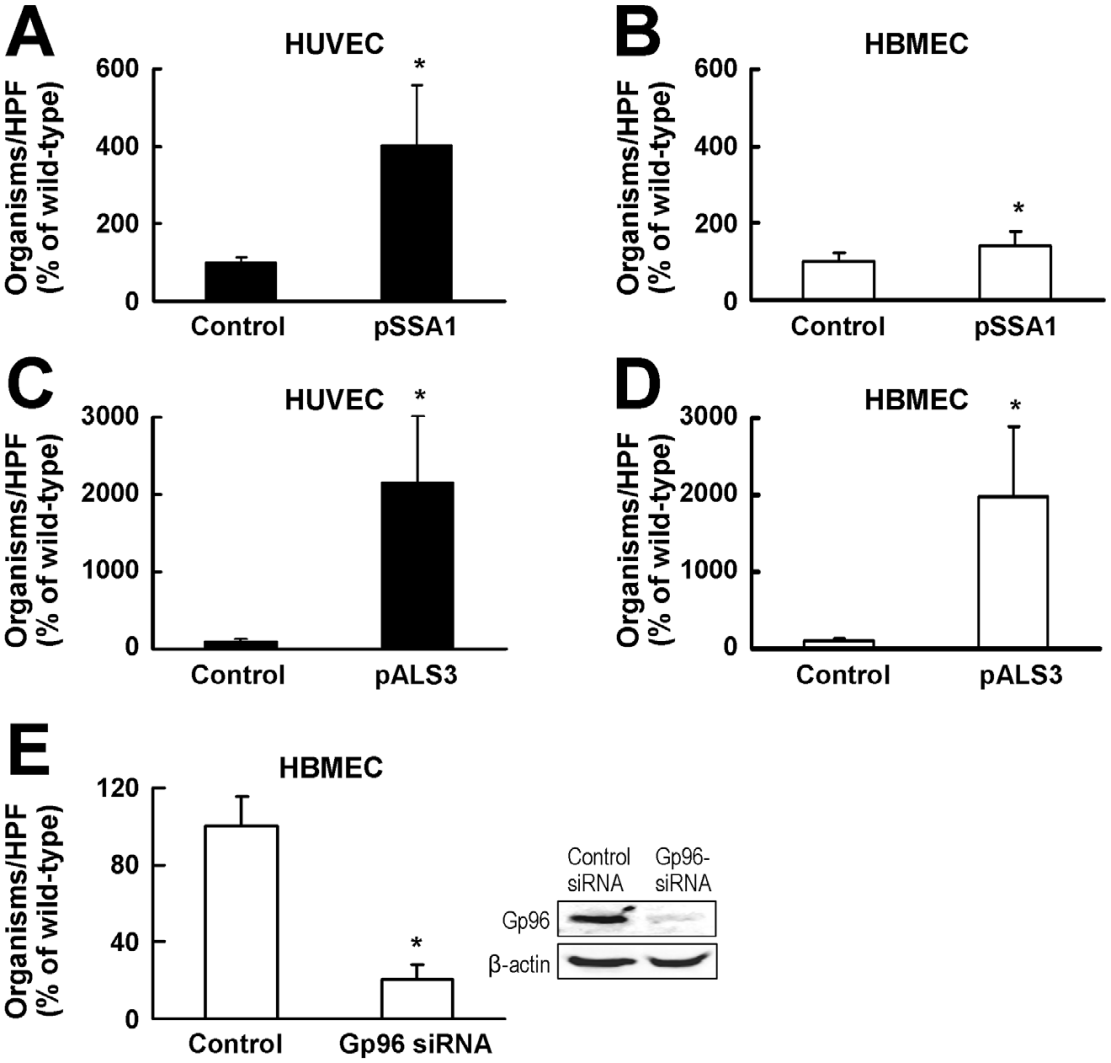
Both Als3 and Ssa1 induce HBMEC endocytosis, but Als3 has a greater effect than Ssa1. (A and B) Endocytosis by HUVECs (A) and HBMECs (B) of *S. cerevisiae* expressing *C. albicans SSA1*, or the backbone vector (control). (C and D) Endocytosis by HUVECs (C) and HBMECs (D) of *S. cerevisiae* expressing *C. albicans* Als3 or the backbone vector (control). (E) Effect of siRNA knockdown of gp96 on HBMEC endocytosis of *S. cerevisiae* expressing *C. albicans ALS3*. Image on the right is of a representative immunoblot of total HBMEC lysates probed for gp96 and β-actin. The endocytosis data are the mean ± SD of 3 experiments, each performed in triplicate. The results in (A–D) are expressed as a percentage of the control strain containing the backbone vector and the results in (E) are expressed as a percentage of the endocytosis of the *ALS3* expressing strain by HBMEC transfected with control siRNA. The mean number of control cells endocytosed by the HUVECs and HBMECs in (A–D) was 3.2 and 1.2 organisms per 10 HPF, respectively. A mean of 49 cells of the *Als3* expressing strain per HPF was endocytosed by HBMEC transfected with the control siRNA in (E). *p<0.01 compared to control strains.

To determine whether Ssa1 and Als3 mediate the endocytosis of *C. albicans* by directly interacting with endothelial cells, we used a heterologous expression strategy in which we expressed *C. albicans SSA1* or *ALS3* in the normally non-invasive yeast, *Saccharomyces cerevisiae* [25]. Expression of *C. albicans SSA1* in *S. cerevisiae* resulted in a 300% increase in the endocytosis of this organism by HUVECs and a 43% increase in its endocytosis by HBMECs, as compared to the control strain of *S. cerevisiae* (Figure 6A and B). Moreover, expression of *C. albicans ALS3* in *S. cerevisiae* resulted in a 2050% and 1880% increase in endocytosis by HUVECs and HBMECs, respectively (Figures 6C and D). Collectively, these data demonstrate that Ssa1 is a more potent inducer of fungal endocytosis by HUVECs than by HBMECs, whereas Als3 can induce endocytosis by HUVECs and HBMECs with similar efficacy. As HUVECs do not express gp96 on their surface [9], HUVEC endocytosis of *S. cerevisiae* expressing *C. albicans SSA1* or *ALS3* is mediated by receptors other than gp96, such as N-cadherin and HER2 [14]–[17].

### Als3 interacts with gp96 to induce HBMEC endocytosis

The above results suggested a model in which Als3 on the surface of *C. albicans* hyphae binds to gp96 on the surface of HBMECs and induces endocytosis. To test this model, we analyzed the effects of siRNA knockdown on the endocytosis of the *S. cerevisiae* strain that expressed *C. albicans* Als3. As predicted, knockdown of gp96 in HBMECs reduced the endocytosis of the Als3 expressing strain of *S. cerevisiae* by 79% compared to control HBMECs (Figure 6E).

**Figure 7:**
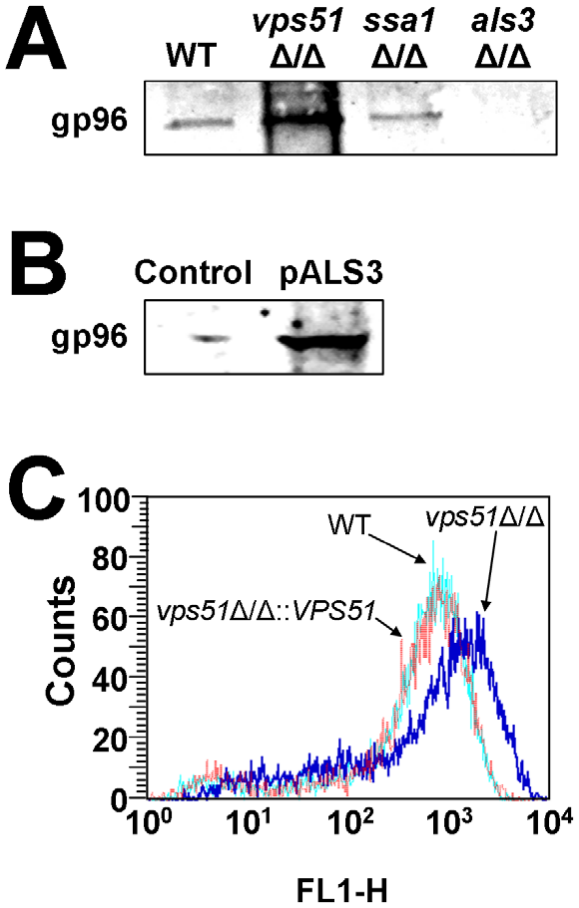
Effects of deletion of *VPS51*, *SSA1*, and *ALS3* on binding to gp96 and fungal surface expression of Als3. (A and B) Immunoblots of HBMEC membrane proteins that were bound by the indicated strains of *C. albicans* (A) or *S. cerevisiae* (B). Both blots were probed with an anti-gp96 antibody. (C) Flow cytometric analysis of Als3 exposure on the surface of hyphae of the wild-type, *vps51*Δ/Δ mutant, and *vps51*Δ/Δ*+*p*VPS51* complemented strains.

We also tested the capacity of different *C. albicans* mutants and strains of *S. cerevisiae* to bind gp96 in HBMEC membrane protein extracts. As predicted by our endocytosis results, the *vps51*Δ/Δ mutant bound more gp96 than did the wild-type strain (Figure 7A). Also, the *ssa1*ΔΔ mutant bound slightly less gp96 than did the wild-type strain, and the *als3*Δ/Δ mutant bound very poorly to this protein. Finally, the strain of *S. cerevisiae* that expressed *C. albicans* Als3 bound to gp96, whereas the control strain of *S. cerevisiae* did not (Figure 7B), thus indicating that Als3 directly interacts with gp96.

Next, we used flow cytometric analysis of *C. albicans* hyphae stained with either anti-HSP70 or anti-Als3 antibodies to quantify the levels of Ssa1 and Als3 that were exposed on the surface of the various strains. Although the *vps51*Δ/Δ mutant had normal Ssa1 surface exposure (data not shown), it had greater surface exposure of Als3 than did the wild-type and *vps51Δ/Δ+*p*VPS51* complemented strains (Figure 7C). The greater surface exposure of Als3 by the *vps51*Δ/Δ mutant likely contributes its enhanced capacity to induced HBMEC endocytosis.

### Ssa1 is important for brain invasion by wild-type *C. albicans* whereas Als3 is necessary for brain invasion by the *vps51*Δ/Δ mutant

**Figure 8:**
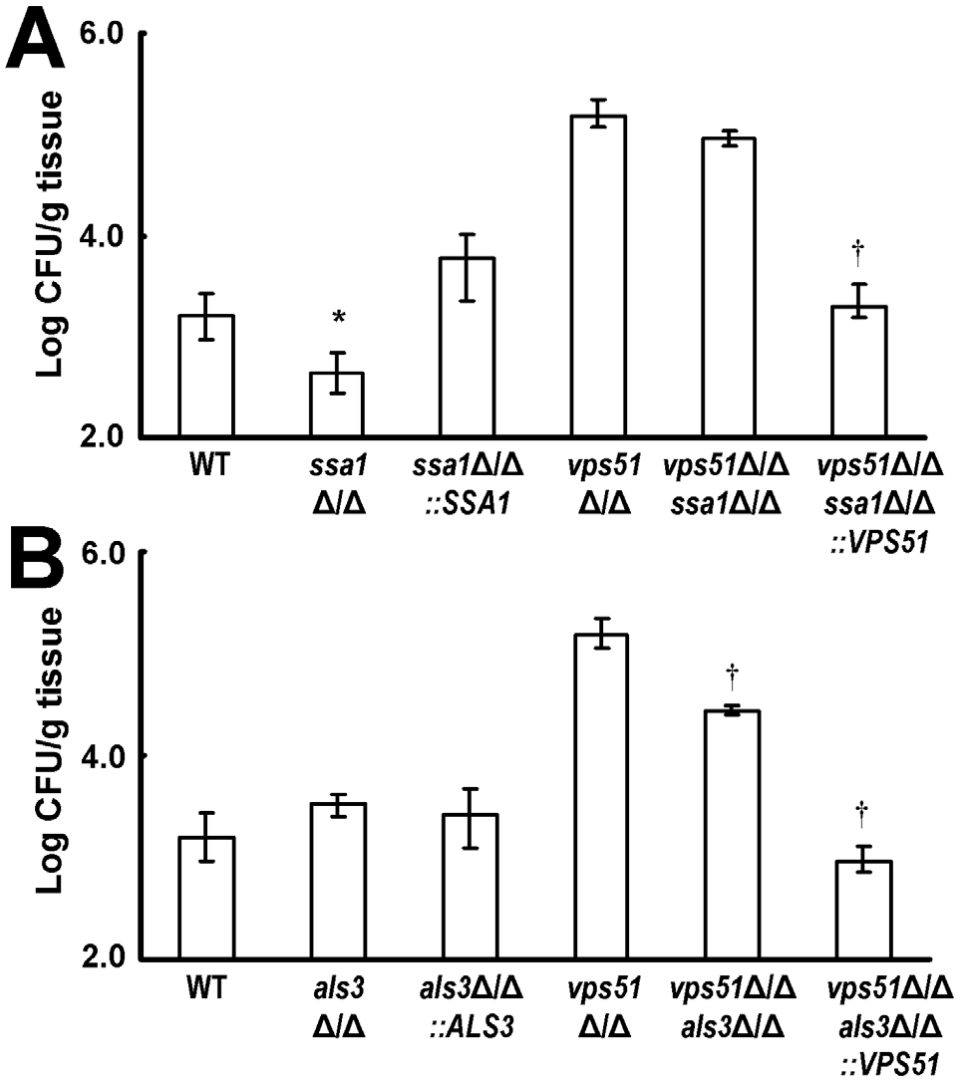
Deletion of *SSA1* and *ALS3* have different effects on brain fungal burden. (A and B) Brain fungal burden of mice after 3 days of infection with the indicated strains of *C. albicans*. Results are median ± interquartile ranges of 7 mice per strain. *p<0.02 compared to mice infected with the wild-type strain; ^†^ *p*<0.001 compared to mice infected with the *vps51*Δ/Δ mutant.

Lastly, we investigated the roles of Ssa1 and Als3 in mediating brain invasion *in vivo* by both wild-type and *vps51*Δ/Δ mutant strains of *C. albicans*. Mice were inoculated with the various *C. albicans* strains via the tail vein and their brain fungal burden was determined 3 days later. Similar to our previous results [14], the brain fungal burden of mice infected with the *ssa1*Δ/Δ single mutant was significantly less than that of mice infected with either the wild-type strain or the *ssa1*Δ/Δ+p*SSA1* complemented strain (Figure 8A). However, the brain fungal burden of mice infected with the *vps51*Δ/Δ *ssa1*Δ/Δ double mutant was only 1.7-fold lower than that of mice infected with the *vps51*Δ/Δ single mutant, a difference that did not achieve statistical significance (*p* = 0.053). Taken together, these results indicate that Ssa1 is necessary for wild-type *C. albicans* to cause maximal brain infection, but that it plays a relatively minor role in enhanced brain tropism of the *vps51*Δ/Δ mutant.

Different results were obtained with strains that lacked Als3. The brain fungal burden of mice infected with the *als3*Δ/Δ single mutant was similar to that of mice infected with the wild-type strain (Figure 8B). In contrast, mice infected with the *vps51*Δ/Δ *als3*Δ/Δ double mutant had 5.5-fold fewer organisms in their brain compared to mice infected with the *vps51*Δ/Δ single mutant. Therefore, although Als3 is dispensable for wild-type *C. albicans* to infect the brain, it is important for the *vps51*Δ/Δ mutant to achieve maximal brain fungal burden.

## Discussion

In the mouse model of disseminated candidiasis, kidney fungal burden is directly correlated with mortality [23], [26]. Thus, many studies of this disease have used kidney fungal burden as the primary endpoint when analyzing either the virulence of mutant strains of *C. albicans* in mice or the susceptibility of mutant strains of mice to disseminated candidiasis [27]–[31]. However, during disseminated candidiasis in both mice and humans, *C. albicans* infects virtually all organs in the body. To do so, the blood-borne organisms must adhere to and invade the vascular beds of these organs. Importantly, there are significant differences among the endothelial cells that line the vasculature of the different organs, as well as the immunologic milieu of these organs [32], [33]. These differences provide a compelling rationale to investigate the capacity of *C. albicans* to traffic to and persist in organs other than the kidney. The brain is a particularly important target organ in neonates with hematogenously disseminated candidiasis [4], [5], and its blood vessels are lined with the unique endothelial cells that form the blood-brain barrier. Our studies with a *vps51*Δ/Δ mutant strain of *C. albicans* led us to discover that *C. albicans* traffics to the brain and invades cerebral blood vessels in part by binding to gp96 that is expressed on the surface of brain endothelial cells.

We had previously identified *C. albicans VPS51* through a microarray study that was designed to discover genes that were up-regulated when the organism adhered to HUVECs [20]. In that study, we determined that a *vps51/vps51* insertion mutant had reduced capacity to damage HUVECs and increased susceptibility to antimicrobial peptides [20]. These *in vitro* findings led us to predict that *VPS51* would be required for the maximal virulence of *C. albicans* during disseminated disease. In the current study, we verified this prediction by determining that mice infected with a *vps51*Δ/Δ deletion mutant had no mortality and progressively cleared this strain from their kidneys and liver.

A unique and unexpected phenotype of the *vps51*Δ/Δ mutant was its marked propensity to infect the brain. In the few previous studies in which the brain fungal burden of mice infected with mutant strains of *C. albicans* was determined, the fungal burden in the brain generally paralleled the fungal burden in the kidney. For example, mice infected with *ecm33*Δ/Δ and *hog1*Δ/Δ mutants had improved survival and reduced fungal burden in both the kidney and the brain, as compared to mice infected with the wild-type strain [34], [35]. Thus, it was unusual to find that mice infected with the *vps51*Δ/Δ mutant had reduced kidney fungal burden, yet significantly increased brain fungal burden.

Our finding that the enhanced capacity of the *vps51*Δ/Δ mutant to adhere to and invade HBMECs, as compared to HUVECs, provides a likely explanation for its brain tropism. One difference between HBMECs and HUVECs is that the former cells express gp96 on their surface, whereas the latter cells do not [9]. Multiple lines of evidence indicate that gp96 functions as an HBMEC receptor for both wild-type *C. albicans* and the *vps51*Δ/Δ mutant. For example, an anti-gp96 antibody and siRNA knockdown of gp96 inhibited HBMEC endocytosis of *C. albicans*. Furthermore, overexpression of gp96 in HBMEC and the heterologous expression of human gp96 in CHO cells increased the endocytosis of *C. albicans*. Finally, wild-type *C. albicans* cells bound to gp96 in extracts of HBMEC membrane proteins, and the highly endocytosed *vps51*Δ/Δ mutant bound even more of this protein. Collectively, these data indicate that gp96 is an HBMEC receptor for *C. albicans*.

It was notable that in both the anti-gp96 antibody studies and the gp96 siRNA experiments, inhibition of gp96 function or expression had greater effect on the endocytosis of the *vps51*Δ/Δ mutant than the wild-type strain (78% reduction for the *vps51*Δ/Δ mutant vs. 38% reduction for the wild-type strain; *p*<0.0001). These results indicate that the *vps51*Δ/Δ mutant preferentially utilizes gp96 as a receptor to invade HBMECs. They further suggest that the enhanced brain tropism of the *vps51*Δ/Δ mutant is likely due to its increased binding to gp96 on the surface of brain endothelial cells.

Although these results strongly indicate that gp96 is important for HBMEC endocytosis of *C. albicans*, the findings that neither the anti-gp96 antibody nor siRNA knockdown of gp96 completely blocked the endocytosis of this organism suggest that it can invade HBMECs by additional mechanisms. Such mechanisms include the induction of endocytosis by binding to one or more receptors, such as N-cadherin that are independent of gp96 and active penetration, in which hyphae physically push their way into host cells by progressively elongating [16], [36].

Because gp96 also functions as a molecular chaperone [37], it is possible that it could be involved in the endocytosis of *C. albicans* by altering the expression or function of other proteins on the surface of HBMECs. Our data indicate that this possibility is remote because HBMEC endocytosis of *C. albicans* was inhibited by the anti-gp96 antibody, which is unlikely to affect the chaperone function of gp96. In addition, siRNA knockdown of gp96 inhibited the endocytosis of *C. albicans* by HBMECs, but not HUVECs, in which gp96 is located intracellularly. Moreover, gp96 knockdown did not affect transferrin uptake in HBMECs, a process that is mediated by the transferrin receptor. Thus, the role of gp96 in inducing the endocytosis of *C. albicans* is likely due to its function as a cell surface receptor rather than a chaperone.

Gp96 has been reported to be expressed on the surface of some epithelial cells where it functions as a receptor for *Listeria monocytogenes*, *Neisseria gonorrhoeae* and bovine adeno-associated virus [38]–[40]. In addition, gp96 on the surface of HBMEC is known to be bound by *E. coli* K1 OmpA [9]. This binding induces the endocytosis of *E. coli* by activating signal transducer and activator of transcription 3 (STAT3), which functions upstream of phosphatidylinositol-3 kinase and protein kinase C-α [41]–[43]. Whether the binding of *C. albicans* to gp96 activates a similar signaling pathway remains to be determined.

*C. albicans* possesses at least two invasin-like proteins, Ssa1 and Als3. Both of these proteins induce the endocytosis of *C. albicans* by HUVECs by binding to N-cadherin and other endothelial cell receptors [14], [15]. These two invasins may function cooperatively because the endocytosis defect of an *ssa1*Δ/Δ *als3*Δ/Δ double mutant is not greater than that of an *als3*Δ/Δ single mutant [14]. Our current studies with the *C. albicans ssa1*Δ/Δ and *als3*Δ/Δ mutants and strains of *S. cerevisiae* that overexpress *C. albicans* Ssa1 and Als3 demonstrate that both of these proteins can induce HBMEC endocytosis. The results of these *in vitro* experiments also indicate that Als3 is more important than Ssa1 in inducing HBMEC endocytosis, probably because it plays a greater role in binding to gp96.

Our mouse studies suggest that Ssa1 is required for the maximal trafficking of wild-type *C. albicans* to the brain because the brain fungal burden of mice infected with the *ssa1*Δ/Δ mutant was significantly less than that of mice infected with the wild-type strain. These results are similar to our previous data [14]. However, deletion of *SSA1* in the *vps51* mutant had only a minor effect on brain trafficking. It is probable that in the *vps51*Δ/Δ mutant, the effects of deleting *SSA1* were masked by the increased surface expression of Als3.

A paradoxical finding was that although the endocytosis of the *als3*Δ/Δ mutant by HBMECs was severely impaired *in vitro*, this mutant had normal trafficking to the brain in mice. The normal virulence of an *als3*Δ/Δ mutant in the mouse model of disseminated candidiasis has recently been reported by others [44]. It is unclear why there is such a large discrepancy between the host cell interactions of the *als3*Δ/Δ mutant *in vitro* and its virulence in mice, especially because *ALS3* is highly expressed *in vivo* [45], [46]. The most probable explanation for these paradoxical results is that other invasins, such as Ssa1 and perhaps other proteins, compensate for the absence of Als3. Because the *in vitro* experiments were performed using human endothelial cells and the virulence experiments were performed in mice, it is theoretically possible that differences between human and mouse gp96 may account for the differences between the *in vitro* and *in vivo* results. However, human and mouse gp96 are 97.5% identical at the amino acid level, making this possibility unlikely.

Importantly, our results indicate that Als3 does play a role in the enhanced brain tropism of the *vps51*Δ/Δ mutant because the brain fungal burden of mice infected with *vps51*Δ/Δ *als3*Δ/Δ double mutant was significantly lower than that of mice infected with the *vps51*Δ/Δ single mutant. Because protein trafficking is likely abnormal in the *vps51*Δ/Δ mutant, we speculate that this strain has reduced expression of compensatory proteins in response to deletion of *ALS3*. On the other hand, the *vps51*Δ/Δ *als3*Δ/Δ double mutant still had greater tropism for the brain compared to the wild-type strain. This result suggests that the overexpression of additional proteins, other than Als3, contributes to the brain tropism of the *vps51*Δ/Δ single mutant.

**Figure 9:**
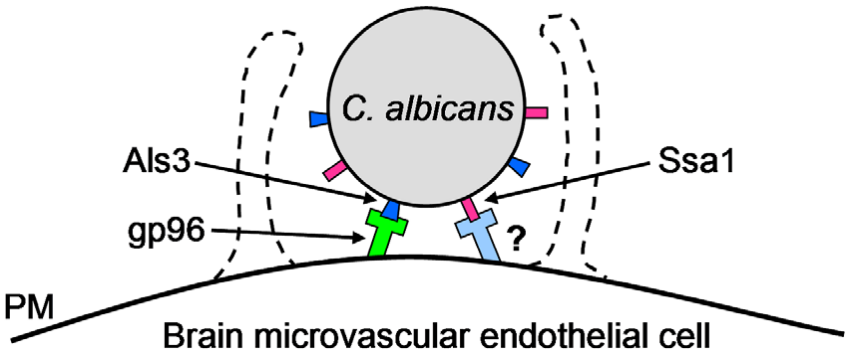
Model of the receptor-ligand interactions that mediate the endocytosis of *C. albicans* by HBMECs. *C. albicans* Als3 binds to gp96 on the surface of HBMECs and induces endocytosis. *C. albicans* Ssa1 binds to an HBMEC surface protein other than gp96, which also induces endocytosis. PM, plasma membrane.

The combined results of these experiments support a model in which *C. albicans* invades the brain during hematogenously disseminated infection by binding to proteins that are specifically expressed on the surface of brain endothelial cells. One of these proteins is gp96, which is bound predominantly by *C. albicans* Als3 (Figure 9). At least one other brain endothelial cell protein functions as receptors for *C. albicans* Ssa1. As the endothelial cells of other vascular beds also express unique surface proteins, it is highly probable that blood-borne *C. albicans* utilizes different endothelial cell surface proteins to infect different organs. Identification of these organ-specific receptors for *C. albicans* may lead to novel approaches to block these receptors and thereby prevent hematogenous dissemination.

## Materials and Methods

### Fungal strains and plasmids

The fungal strains used in this study are listed in Supplemental Table S1. All C. albicans mutant strains constructed for this study were derived from strain BWP17 [47]. Deletion of the entire protein coding regions of both alleles of *VPS51* was accomplished by successive transformation with *ARG4* and *HIS1* deletion cassettes that were generated by PCR using the oligonucleotides vps51-f and vps51-r (The oligonucleotide sequences are listed in Supplemental Table S2) [47]. The resulting strain was subsequently transformed with pGEM-URA3 [47] to re-integrate *URA3* at its native locus. The *vps53*Δ/Δ mutant was constructed similarly, using the oligonucleotides vps53-f and vps53-r. To construct the *VPS51* complemented strain (*vps51*Δ/Δ*+*p*VPS51*), a 2.6 Kb fragment containing VPS51 was generated by high fidelity PCR with the primers vps51-rev-f and vps51-rev-r using genomic DNA from C. albicans SC5314 as the template. This PCR product was digested with *Nco*I, and then subcloned into pBSK-Ura, which had been linearized with NcoI. The resulting construct was linearized with NotI and *Pst*I to direct integration at the URA3 locus of a Ura^–^ *vps51*Δ/Δ mutant strain. The *vps53*Δ/Δ-complemented strain (*vps53*Δ/Δ+p*VPS53*) was generated similarly, except that primers vps53-rev-f and vps53-rev-r were used to PCR amplify a 3.3 Kb DNA fragment containing *VPS53*.

To delete the entire protein coding region of ALS3 in the *vps51*Δ/Δ mutant, deletion cassettes containing ALS3 flanking regions and the URA3 or NAT1 selection markers were amplified by PCR with primers als3-pgem-KO-f and als3-pgem-KO-r, using pGEM-URA3 [47] and pJK795 [48] as templates, respectively. These PCR products were then used to successively transform a Ura- *ssa1*Δ/Δ strain. The resulting als3Δ/Δ vps51Δ/Δ double mutant was plated on 5-fluoroorotic acid to select for a Ura- strain, which was then transformed with pGEM-URA3 as above. The als3Δ/Δ vps51Δ/Δ*+*p*VPS51* complemented strain was generated the same way as was the vps51Δ/Δ*+*p*VPS51* complemented strain. The ssa1Δ/Δ vps51Δ/Δ double mutant and its *VPS51*-complemented strain (*ssa1*Δ/Δ vps51Δ/Δ*+*p*VPS51*) were generated similarly to the als3Δ/Δ vps51Δ/Δ double mutant and its complemented strain, except that primers ssa1-pgem-f and ssa1-pgem-r were used to amplify the *SSA1* deletion cassettes.

The construction of the *S. cerevisiae* strain that expressed *C. albicans ALS3* under the control of the *ADH1* promoter and its control strain containing the backbone vector was described previously [25]. To express *C. albicans SSA1* in *S. cerevisiae*, a 2.0 kb fragment containing the *SSA1* protein coding region was generated by PCR with primers ssa1-exp-bglii-f and ssa1-exp-xhoi-r using pRP10-SSA1ORF as template [49]. The resulting *SSA1* fragment was cloned downstream of the *GAL1* promoter of pYES2.1/V5-His-TOPO using the pYES2.1 TOPO TA Expression Kit (Invitrogen) following the manufacturer's instructions. The control strain of *S. cerevisiae* was transformed with the backbone vector alone. Expression of *C. albicans SSA1* was induced by growth in SC minimal medium containing 2% galactose following the manufacturer's protocol.

### Murine model of disseminated candidiasis

Male BALB/c mice weighing 18–20 g (Taconic Farms) were used for all animal experiments. For survival studies, 10 mice per strain were injected via the tail vein with either 5×10^5^ or 3×10^6^ yeast of the various C. albicans strains [50] and then monitored for survival three times daily. All inocula were confirmed by colony counting. In the organ fungal burden studies, the mice were inoculated with 5×10^5^ yeast as above. At various time points, 7 mice per strain were sacrificed and the kidney, liver, and brain were harvested. These organs were weighed, homogenized and quantitatively cultured. For histopathological analysis, a portion of the excised tissue was fixed in zinc-buffered formalin followed by 70% ethanol. The tissue was then embedded in paraffin, after which thin sections were prepared and stained with Gomori methenamine silver. They were examined by light microscopy. All mouse experiments were approved by the Animal Care and Use Committee at the Los Angeles Biomedical Research Institute and carried out according to the National Institutes of Health (NIH) guidelines for the ethical treatment of animals.

### Endothelial cells

HUVECs were harvested from umbilical cords with collagenase and grown in M-199 medium supplemented with 10% fetal bovine serum and 10% defined bovine calf serum (Gemini Bio-Products), and containing 2 mM L-glutamine with penicillin and streptomycin (Irvine Scientific) as previously described [51]. HBMECs were isolated from the capillaries in small fragments of the cerebral cortex, which were obtained by surgical resection from 4- to 7-year-old children with seizure disorders at Children's Hospital Los Angeles. HBMECs were harvested from these capillaries and maintained in a mixture of M-199 and Ham's F-12 media (1∶1 v/v) supplemented with 10% fetal bovine serum, 1 mM sodium pyruvate, and 2 mM glutamine as described previously [52]. More than 98% of these cells were positive for Factor VIII-rag and carbonic anhydrase, and negative for GFAP by flow cytometry. In addition, 99% of the cells took up Dil-Ac-LDL by immunocytochemistry. CHO K-1 cells expressing human gp96 were generated and grown as outlined before [9]. All cell types were grown at 37°C in 5% CO_2_.

### Knockdown of gp96 by siRNA

HBMECs or HUVECs were grown to 60% confluence in six-well plates, then transfected with either gp96 siRNA (Catalog number HSS110955; Invitrogen) or a random control siRNA using lipofactamine 2000 (Invitrogen), according to the manufacturer's instructions. Gp96 knockdown was verified by Western blotting of total endothelial cell lysates with an anti-gp96 monoclonal antibody (Santa Cruz Biotechnology).

### Affinity purification of gp96 using intact organisms

HBMEC membrane proteins from host cells were isolated using octyl-glucopyranoside exactly as described previously [16]. Next, 2×10^8^ hyphae of the various *C. albicans* strains or 8×10^8^ yeast of the different *S. cerevisiae* strains were incubated on ice for 1 h with 250 µg of HBMEC membrane proteins in PBS with calcium and magnesium and containing 1.5% octyl-glucopyranoside and protease inhibitors. The unbound proteins were removed by extensive rinsing in the same buffer. Next, the proteins that had bound to the hyphae were eluted with 6M urea. The eluted proteins were separated by SDS-PAGE and detected by immunoblotting with the anti-gp96 antibody using enhanced chemiluminescence (Pierce).

### Candidal adherence

The adherence of *C. albicans* to HUVECs and HBMECs grown in 6-well tissue culture plates was measured by a modification of our previously described method [25]. Briefly, germ tubes of the various strains were generated by a 1-h incubation in RPMI 1640 medium (Irvine Scientific) at 37°C. The germ tubes were enumerated with a hemacytometer and suspended in HBSS at 200 cells/ml. After rinsing the endothelial cell monolayers twice with HBSS, 1 ml of the germ tube suspension was added to each well. The cells were incubated for 30 min, after which the nonadherent organisms were aspirated and the endothelial cell monolayers were rinsed twice with HBSS in a standardized manner. Next, the wells were overlaid with YPD agar and the number of adherent organisms was determined by colony counting. The adherence results were expressed as a percentage of the initial inoculum, which was verified by quantitative culture. Each strain was tested in triplicate on three different days.

### Candidal endocytosis

The number of organisms internalized by the endothelial cells was determined using our standard differential fluorescence assay [15], [16]. Briefly, endothelial cells on glass coverslips were infected with 10^5^ yeast phase cells of each strain of *C. albicans* in RPMI 1640 medium. After incubation for 3 h, the cells were fixed with 3% paraformaldehyde. The noninternalized cells were stained with anti-*C. albicans* rabbit serum (Biodesign International) that had been conjugated with Alexa 568 (Invitrogen). Next, the endothelial cells were permeablized in 0.1% (vol/vol) Triton X-100 in PBS, after which both the internalized and the noninternalized organisms were stained with anti-*C. albicans* rabbit serum conjugated with Alexa 488 (Invitrogen). The coverslips were mounted inverted on a microscope slide and viewed under epifluorescence. The number of organisms endocytosed by the endothelial cells was determined by subtracting the number of noninternalized organisms (which fluoresced red) from the total number of organisms (which fluoresced green). At least 100 organisms were counted on each coverslip, and all experiments were performed in triplicate on at least three separate occasions.

### Transferrin uptake

HBMECs were grown to 70% confluency in 6-well tissue culture plates and then incubated for 3 in serum-free medium to deplete endogenous transferrin. Next they were incubated for 45 min in serum-free medium containing AlexaFluor 555-labeled transferrin (Invitrogen; 10 µg/ml). The unincorporated transferrin was removed by rinsing, after which the cells were incubated for an additional 30 min. Any remaining surface bound transferrin was removed by rinsing the cells twice with ice-cold PBS containing Ca^++^ and Mg^++^ (PBS^++^) followed by two, 5 min incubations with ice-cold acid wash buffer (0.2 M acetic acid (pH 2.8) 0.5 M NaCl). Finally, the cells were washed three times with ice-cold PBS^++^, detached with Cell Dissociation Buffer (Invitrogen), and suspended in PBS^++^. Their transferrin content was determined by flow cytometry, analyzing at least 10,000 cells.

### Flow cytometry

Flow cytometry was used to analyze the surface expression Als3p on hyphae of the various strains using a minor modification of our previously described method [14]. Briefly, hyphae of the different strains of *C. albicans* were fixed in 3% paraformaldehyde and blocked with 1% goat serum. The hyphae were then incubated with either a rabbit polyclonal antiserum raised against rAls3-N or purified rabbit IgG. After extensive rinsing, the cells were incubated with a goat anti-rabbit secondary antibody conjugated with Alexa 488. The fluorescent intensity of the hyphae was measured by flow cytometry. Fluorescence data for 10,000 cells of each strain were collected.

### Statistical analyses

The capacity of the various strains of C. albicans and *S. cerevisiae* to adhere to, and be endocytosed to endothelial cells was compared using analyses of variance. Differences in the fungal burden of mice infected with these strains were analyzed using the Wilcoxon Rank Sum test. Differences in survival were analyzed using the Log-Rank test.

### Ethics statement

The protocol for collecting umbilical cords for the harvesting of HUVECs used in these studies was approved by the Institutional Review Board of the Los Angeles Biomedical Research Institute at Harbor-UCLA Medical Center. This protocol was granted a waiver of consent because the donors remained anonymous. The protocol for using fragments of the cerebral cortex, obtained by surgical resection from 4- to 7-year-old children with seizure disorders, for isolation of HBMECs was approved by the Institutional Review Board of Childrens Hospital Los Angeles. These fragments were obtained from anonymous donors in 1992-1993 and the HBMECs used in the current studies were isolated at that time and stored in liquid nitrogen. The use of HBMECs in our studies is exempted because the donors are unknown and there is no information linking the HBMECs with the donors. The mouse studies were carried out in accordance with the National Institutes of Health guidelines for the ethical treatment of animals. This protocol was approved by the Institutional Animal Care and Use Committee (IACUC) of the Los Angeles Biomedical Research Institute at Harbor-UCLA Medical Center.

## Supporting Information

Table S1 List of fungal strains used in this study and their relevant genotypes.
(DOC)

Table S2 List of PCR primers used to construct the C. albicans mutants and verify their genotypes.
(DOC)
