# Supplementary material for: Mechanisms of Candida albicans Trafficking to the Brain
Source: PLoS Pathog. 2011 Oct 6;7(10):e1002305. doi: 10.1371/journal.ppat.1002305 (PMC3188548; doi:10.1371/journal.ppat.1002305)
Supplement: Table S1 — List of fungal strains used in this study and their relevant genotypes. (DOC) [file ppat.1002305.s001.doc]

Table S1. Fungal strains used in this study.

| **Strain** | **Genotype** | **Source [Reference]** |
| --- | --- | --- |
| **C. albicans** | | |
| BWP17 | *ura3*Δ::*λimm434/ura3*Δ::*λimm434*  *arg4::hisG/arg4::hisG his1::hisG/his1::hisG* |  |
| DAY185 | *ura3*Δ::*λimm434/ura3*Δ::*λimm434*  *arg4::hisG::ARG4::URA3/arg4::hisG*  *his1::hisG::HIS1/his1::hisG* |  |
| CAYF178U | *als3::ARG4/als3::HIS1*  *ura3*Δ*::λimm434::URA3-IRO1/ura3*Δ*::λimm434 arg4::hisG/ arg4::hisG his1::hisG/his1::hisG* |  |
| CAQTP178U | *als3::ARG4::ALS3/als3::HIS1 ura3*Δ*::λimm434::URA3-IRO1/ura3*Δ*::λimm434 arg4::hisG/arg4::hisG his1::hisG /his1::hisG* |  |
| *ssa1*Δ/Δ-*URA3* | *ura3*Δ::*λimm434/ura3*Δ::*λimm434* ssa1Δ::FRT/ssa1Δ::FRT SSA2/ssa2::FRT rps10::URA3/RPS10 |  |
| *ssa1*Δ/Δ+*SSA1* | *ura3*Δ::*λimm434/ura3*Δ::*λimm434* ssa1Δ::FRT/ssa1Δ::FRT SSA2/ssa2::FRT rps10::URA3::SSA1/RPS10 |  |
| *vps51*Δ/Δ | *vps51*Δ*::HIS1/vps51*Δ*::ARG4 ura3*Δ*::λimm434::URA3/ura3*Δ*::λimm434* *arg4::hisG/arg4::hisG his1::hisG/his1::hisG* | This study |
| *vps51*Δ/Δ-  complement | *vps51*Δ*::HIS1/vps51*Δ*::ARG4 ura3*Δ*::λimm434::URA3::VPS51/ura3*Δ*::λimm434* *arg4::hisG/arg4::hisG his1::hisG/his1::hisG* | This study |
| *vps51*Δ/Δ-*als3*Δ/Δ | *vps51*Δ*::HIS1/vps51*Δ*::ARG4*  *als3*Δ::*ura3/als3*Δ*::NAT1 ura3*Δ*::λimm434::URA3/ura3*Δ*::λimm434* *arg4::hisG/arg4::hisG his1::hisG/his1::hisG* | This study |
| *vps51*Δ/Δ-*als3*Δ/Δ-  complement | *vps51*Δ*::HIS1/vps51*Δ*::ARG4*  *ssa1*Δ::*ura3/ssa1*Δ*::NAT1 ura3*Δ*::λimm434::URA3::VPS51/ura3*Δ*::λimm434* *arg4::hisG/arg4::hisG his1::hisG/his1::hisG* | This study |
| *vps51*Δ/Δ-*ssa1*Δ/Δ | *vps51*Δ*::HIS1/vps51*Δ*::ARG4*  *ssa1*Δ::*ura3/ssa1*Δ*::NAT1 ura3*Δ*::λimm434::URA3/ura3*Δ*::λimm434* *arg4::hisG/arg4::hisG his1::hisG/his1::hisG* | This study |
| *vps51*Δ/Δ-*ssa1*Δ/Δ-  complement | *vps51*Δ*::HIS1/vps51*Δ*::ARG4*  *ssa1*Δ::*ura3/ssa1*Δ*::NAT1 ura3*Δ*::λimm434::URA3::VPS51/ura3*Δ*::λimm434* *arg4::hisG/arg4::hisG his1::hisG/his1::hisG* | This study |
| *vps53*Δ/Δ | *vps53*Δ*::HIS1/vps53*Δ*::ARG4 ura3*Δ*::λimm434::URA3/ura3*Δ*::λimm434* *arg4::hisG/arg4::hisG his1::hisG/his1::hisG* | This study |
| *vps53*Δ/Δ-  complement | *vps53*Δ*::HIS1/vps53*Δ*::ARG4 ura3*Δ*::λimm434::URA3::VPS53/ura3*Δ*::λimm434* *arg4::hisG/arg4::hisG his1::hisG/his1::hisG* | This study |
| ***S. cerevisiae*** | | |
| S150-2B | *leu2 his3 trp1 ura3* |  |

**References**

1. Wilson RB, Davis D, Mitchell AP (1999) Rapid hypothesis testing with *Candida albicans* through gene disruption with short homology regions. J Bacteriol 181: 1868-1874.

2. Davis D, Wilson RB, Mitchell AP (2000) *RIM101*-dependent and-independent pathways govern pH responses in *Candida albicans*. Mol Cell Biol 20: 971-978.

3. Nobile CJ, Andes DR, Nett JE, Smith FJ, Yue F, et al. (2006) Critical role of Bcr1-dependent adhesins in *C. albicans* biofilm formation in vitro and in vivo. PLoS Pathog 2: e63.

4. Sun JN, Solis NV, Phan QT, Bajwa JS, Kashleva H, et al. (2010) Host cell invasion and virulence mediated by *Candida albicans* Ssa1. PLoS Pathog 6: e1001181.

5. Li XS, Sun JN, Okamoto-Shibayama K, Edgerton M (2006) *Candida albicans* cell wall Ssa proteins bind and facilitate import of salivary histatin 5 required for toxicity. J Biol Chem 281: 22453-22463.

6. Fu Y, Filler SG, Spellberg BJ, Fonzi W, Ibrahim AS, et al. (1998) Cloning and characterization of *CAD1/AAF1*, a gene from *Candida albicans* that induces adherence to endothelial cells after expression in *Saccharomyces cerevisiae*. Infect Immun 66: 2078-2084.
