# Supplementary material for: Mechanisms of Candida albicans Trafficking to the Brain
Source: PLoS Pathog. 2011 Oct 6;7(10):e1002305. doi: 10.1371/journal.ppat.1002305 (PMC3188548; doi:10.1371/journal.ppat.1002305)
Supplement: Table S2 — List of PCR primers used to construct the C. albicans mutants and verify their genotypes. (DOC) [file ppat.1002305.s002.doc]

Table S2. PCR primers used to construct the *C. albicans* mutants

| Primer Name | Primer sequence (5’ to 3’) |
| --- | --- |
| vps51-f | CCAGATATACGAGCGCTCATAAGAGAAGAACAATCAAGAAATCAAGA  ACTCAATTCACGTTTAT TCCAAGCTTCCTGTGGAATTGTGAGCGGATA |
| vps51-r | ATCAGGCAAGAAATTTAAAAATCATTCAGTTGATGATATTCTATAAATA  TATCTATAA ATGCGTAGGTAGATATGAATTTCCCAGTCACGACGTT |
| vps53-f | GAGATCCGTAGCGGTTATGTAAGATTGGTGGGGCAATAAAGGTGTAA  ACAA AACTATGTTGTTGTGTAGAAAATGTGGAATTGTGAGCGGATA |
| vps53-r | CATCTCGACCAACTTGTCGTTAAAATTACGCACATAGCTATCCCTGAT  AATGAG CGGAAGAATCACCCTCAAGTTTCCCAGTCACGACGTT |
| vps51-rev-f | GTTGCCATGGCTGTCTTTGTCGTCTTC |
| vps51-rev-r | CTACCCATGGAAAACACCCAACCAACC |
| vps53-rev-f | CAATAGATGCGGCTGCGTGTC |
| vps53-rev-r | GGAACAACATACCTGGGAATTAACC |
| als3-pgem-ko-f | GATATTTTGAATATGGAAATAAATCGTGCATAAGAAAGTTTTGCTAT  GCACGTTCATACTTCCAAAAATTGTAATACGACTCACTATAGGGC |
| als3-pgem-ko-r | AAACTATAGAAACAAACTAATCAAATTAACAACACACCAAATTGG  AGGTAATTAATCATACCGAAAATAGCTATGACCATGATTACGCCA |
| ssa1-pgem-ko-f | ATTTTCCAATTTTTTCAATTTATTTTATTTTATTCTATTCTATTCTATTCTA  TTCTTCTTTACTTTTTCTTTAATACGACTCACTATAGGGC |
| ssa1-pgem-ko-r | AGAAATAAAAACTAAAATAAAAACCTTTTAAAATTAAACTAAAAAAC  CAACTCCACAGTAAATTACCTCAGCTATGACCATGATTACGCCA |
